# Antibiotic Discovery and Resistance: The Chase and the Race

**DOI:** 10.3390/antibiotics11020182

**Published:** 2022-01-30

**Authors:** Katia Iskandar, Jayaseelan Murugaiyan, Dalal Hammoudi Halat, Said El Hage, Vindana Chibabhai, Saranya Adukkadukkam, Christine Roques, Laurent Molinier, Pascale Salameh, Maarten Van Dongen

**Affiliations:** 1 Department of Mathématiques Informatique et Télécommunications, Université Toulouse III, Paul Sabatier, INSERM, UMR 1295, 31000 Toulouse, France; 2 INSPECT-LB: Institut National de Santé Publique, d’Épidémiologie Clinique et de Toxicologie-Liban, Beirut 6573, Lebanon; pascalesalameh1@hotmail.com; 3 Faculty of Pharmacy, Lebanese University, Beirut 6573, Lebanon; 4 Department of Biological Sciences, SRM University–AP, Amaravati 522502, India; jayaseelan.m@srmap.edu.in (J.M.); saranya_shekharan@srmap.edu.in (S.A.); 5 Department of Pharmaceutical Sciences, School of Pharmacy, Lebanese International University, Bekaa Campus, Beirut 1103, Lebanon; 6 Faculty of Medicine, Lebanese University, Beirut 6573, Lebanon; saidelhage2020@gmail.com; 7 Division of Clinical Microbiology and Infectious Diseases, School of Pathology, University of the Witwatersrand, Johannesburg 2193, South Africa; vindana.chibabhai@nhls.ac.za; 8 Microbiology Laboratory, National Health Laboratory Service, Charlotte Maxeke Johannesburg Academic Hospital, Johannesburg 2193, South Africa; 9 Laboratoire de Génie Chimique, Department of Bioprocédés et Systèmes Microbiens, Université Paul Sabtier, Toulouse III, UMR 5503, 31330 Toulouse, France; roques730@aol.com; 10 Department of Medical Information, Centre Hospitalier Universitaire, INSERM, UMR 1295, Université Paul Sabatier Toulouse III, 31000 Toulouse, France; molinier.l@chu-toulouse.fr; 11 Department of Primary Care and Population Health, University of Nicosia Medical School, Nicosia 2408, Cyprus; 12 AMR Insights, 1017 EG Amsterdam, The Netherlands; info@amr-insights.eu

**Keywords:** antibiotics, antimicrobial resistance, innovative antibiotics

## Abstract

The history of antimicrobial resistance (AMR) evolution and the diversity of the environmental resistome indicate that AMR is an ancient natural phenomenon. Acquired resistance is a public health concern influenced by the anthropogenic use of antibiotics, leading to the selection of resistant genes. Data show that AMR is spreading globally at different rates, outpacing all efforts to mitigate this crisis. The search for new antibiotic classes is one of the key strategies in the fight against AMR. Since the 1980s, newly marketed antibiotics were either modifications or improvements of known molecules. The World Health Organization (WHO) describes the current pipeline as bleak, and warns about the scarcity of new leads. A quantitative and qualitative analysis of the pre-clinical and clinical pipeline indicates that few antibiotics may reach the market in a few years, predominantly not those that fit the innovative requirements to tackle the challenging spread of AMR. Diversity and innovation are the mainstays to cope with the rapid evolution of AMR. The discovery and development of antibiotics must address resistance to old and novel antibiotics. Here, we review the history and challenges of antibiotics discovery and describe different innovative new leads mechanisms expected to replenish the pipeline, while maintaining a promising possibility to shift the chase and the race between the spread of AMR, preserving antibiotic effectiveness, and meeting innovative leads requirements.

## 1. Introduction

A sharp decline in the introduction of newer antibiotics and the worldwide emergence of antimicrobial resistance (AMR), combined with the current COVID-19 pandemic, point to the urgency for action plans for the better treatment of infectious microorganisms [1,2]. Maintaining antibiotic effectiveness and investment towards newer antibiotics discovery may contribute favorably to the global initiatives and implemented programs to fight against AMR [3,4]. It is time to shift the AMR research paradigm for innovation in antibiotic discovery, though this alone cannot be the only solution to this silent pandemic [5,6,7,8]. The spread of AMR is outpacing almost every counter measure, and the world has limited choices to treat infections [9]. Curbing AMR requires complex and specific multi-sectoral measures [10,11,12]. The World Health Organization (WHO) report indicates a discovery void wherein a limited number of new leads are innovative [11,13]. Most of the drugs in the clinical pipeline are intended to act on the same targets as traditional antibiotics [13,14,15,16]. The challenges for antibiotics discovery are also beyond technical and economic limits. Multiple public, private, and public–private initiatives are undertaken to incentivize the research and development of new antibiotics, but are they efficiently prioritized [3,17,18,19]? Here, we aim to describe the history of antibiotics and antimicrobial resistance, and the various innovative new leads and related mechanisms of action expected to replenish the pipeline and shift the chase and the race between the spread of AMR and innovative antibiotics discovery.

## 2. Antimicrobial Resistance Is Ancient

### 2.1. The Environmental Resistome and Its Relation to Antimicrobial Resistance

AMR is an ancient natural widespread phenomenon that precedes humanity [20,21]. Different genetic and biochemical pathways involving multiple facets of bacterial cell function may be the leading cause of AMR. The environmental resistome can potentially provide answers about the origins of AMR, where multi-drug resistant (MDR) bacterial species and modern resistant genes were found in archaeological samples [22]. By definition, the environmental resistome is the whole collection of genes that directly or indirectly contribute to antibiotic resistance. Nowadays, enough evidence has accumulated to show that the environment is the largest source and most significant reservoir of resistance. Soil, aquatic, atmospheric, animal-associated, and built ecosystems are home to microbes that include AMR elements and the genetic means to mobilize them. The diversity and abundance of resistance in the environment is consistent with ancient sources of antibiotics, and studies do support a long natural history of associated resistance. A targeted metagenomic analysis of 30,000-year-old Beringian permafrost samples identified resistance genes to β-lactams, tetracyclines, and glycopeptides antibiotics [21,22]. The genome comparison between modern isolates and 2.7 million years of permafrost strains samples showed no significant differences in antibiotic resistance profiles between ancient and currently studied pathogens [20,21]. A phylogenetic analysis of three million years of permafrost obtained at Mammoth Mountain in Siberia showed that OXA genes encoding β-lactamases have been on plasmids for a million years [23]. AMR determinants were found in natural environments in undisturbed pristine soil within the Mackay glacier, a region naïve to anthropogenic antibiotic use. An analysis of the soft tissue of frozen mummified humans from the copper age showed the presence of resistant genes to β-lactams and glycopeptides that colonized ancient human hosts [24]. Evidence of the high diversity of resistance determinants in antibiotic–naïve ecosystems and the postulated wide spectrum of the intrinsic resistome show that elements contributing to antibiotic resistance can be independent of the anthropogenic use of antibiotics [25], and may not be the result of horizontal gene transfer [26,27,28,29,30]. Resistant determinants found in producers, non-producers, soil, and environmental bacteria are more genetically and mechanistically diverse than what has emerged in pathogens [31,32]. Once mobilized and transferred in the pool of humans and animals, they provided significant opportunities for the exchange and dissemination of resistance [33]. The metagenomic and sequencing techniques allow one to examine the resistant genes in multiple ecosystems (the environmental and human commensal microbiota) and the mechanisms of the acquisition of resistant determinants by human pathogens [29]. Sampling the resistome may provide an early warning of potential future emerging bacterial resistance in clinical isolates [21].

### 2.2. Resistance, Tolerance and Persistence

Resistance is the inherited ability of a bacterium to grow when exposed to high concentrations of antibiotics [34]. Antibiotic susceptibility testing (AST) is a laboratory procedure used to evaluate bacterial resistance. It can be either a genotypic test that determines resistance based on the availability of specific resistant genes, or a phenotypic test that uses either a broth micro-dilution (BMD) or a disk diffusion assay [35,36]. The genotypic test is expensive and limited to a defined array of genes, while the phenotypic test is the reference standard testing used in clinical practice [35]. The BMD provides a semi-quantitative measurement of the minimum inhibitory concentration (MIC) for an antibiotic [35]. MIC allows the estimation of the lowest concentration of antibiotics that inhibits bacterial growth, usually within an average of 16–20 h [36]. MIC does not necessarily correctly predict the clinical effectiveness of the antibiotic in vivo [37].

Tolerance is the ability of a bacterium to survive exposure to increased antibiotic concentrations without modifications of the MIC, as a result of slowing down essential bacterial processes [38,39]. Tolerance can be acquired through genetic mutation or conferred by environmental stress conditions [39]. Unlike resistance, tolerance applies to bactericidal and not to bacteriostatic antibiotics [38], and develops through two pathways:

1. Tolerance by slow growth that is either inherited or not, occurring at a steady state.
2. Tolerance by lag that is a transient state induced by starvation or stress.

Prolonged exposure to an antibiotic rather than a higher concentration of an antibiotic is needed to kill tolerant bacteria [39]. The minimum duration for killing (MDK_99_) is a technique used to measure tolerance, and is defined as the time needed to kill 99% of the culture [39].

The terms ‘resistance’ and ‘tolerance’ are attributable to a whole bacterial population, while persistence refers to a subpopulation of the clonal bacterial population, tolerant to antibiotic treatment, but remaining dormant and metabolically inactive [34].

### 2.3. Intrinsic, Phenotypic and Acquired Resistance

The intrinsic resistome is defined as the set of elements that contribute either directly or indirectly to antibiotic resistance, independently of previous antibiotic exposure, and not due to horizontal gene transfer [26,27]. The classic determinants of intrinsic resistance are changes in bacterial permeability, antibiotic inactivation, and target modification [27,40]. Phenotypic resistance is a non-inheritable resistance in which a susceptible bacterial population becomes transiently resistant. The elements contributing to this phenotype are part of the intrinsic resistome and are only detected in specific growing conditions. Phenotypic resistance can occur through different processes that include persistence, growth in biofilms, and swarming adaptation [27]. Acquisition of resistance can occur through the mutation of these elements and the acquisition of a resistance gene by horizontal gene transfer (HGT). The mechanisms of acquired resistance also include antibiotic inactivation, target modification, and antibiotic efflux [40].

#### 2.3.1. Intrinsic Resistance

Intrinsic antibiotic resistance is a trait within the genome of the bacterial species [41], independent of previous antibiotic exposures, and is not related to HGT [40]. The mechanisms of intrinsic resistance are fixed in the core genetic make-up of the microorganism, and are normally chromosome-encoded [42]. The most common ones include non-specific active efflux pumps that may have actively evolved as a response to environmental toxins [42], such as the AcrAB/TolC efflux pump in *Escherichia coli* [43], and also limited outer membrane permeability, such as the vancomycin resistance in *E.coli* [26,41,42,43,44,45]. Studies have shown that additional genes and genetic loci appear to contribute to this phenotype [41]. Gram-negative bacteria (GNB) are known to be more intrinsically resistant than Gram-positive bacteria (GPB). The treatment of MDR-GNB is challenging due to the presence of an outer membrane permeability barrier to antibiotic influx or to multiple MDR efflux pumps that reduce the intracellular concentration of antibiotics [42]. Intrinsic mechanisms usually confer a low level of resistance in the original host. In immunocompromised patients, intrinsically resistant normal commensal flora or environmental bacteria can become opportunistic [42,45].

#### 2.3.2. Phenotypic Resistance

Phenotypic resistance is a situation in which susceptible bacteria become transiently resistant, either by the development of persistence, or growth in biofilms, or swarming adaptation [27]. Persisters are a subpopulation of bacteria that enter a dormant state, stop actively growing, and enter a stationary growth phase [45]. Persistent cells may be the major contributor to chronic infections, are not the consequence of genetic changes [27,40,46], occur at a rate of 1% in culture, and do not possess resistant genes. Compared with non-persisters, they can survive a longer period after exposure to high antibiotic concentrations [47], resulting in a biphasic killing curve [34], owing to the heterogeneity of the response among the two subpopulations [39]. Biofilm is a complex structure of bacterial colonies encased in a polymer matrix of polysaccharides, proteins, and extracellular DNA [27]. Biofilms confer the bacteria up to 1000 times more antibiotic resistance than planktonic bacteria (free-swimming) [27]. The planktonic form characterizes acute infections, and the biofilm mode is the leading cause of both chronic and device-related infections [45].

Bacterial biofilm communities have a great ability to tolerate antibiotics, host immune defense systems, shear forces, and harsh conditions, leading to MDR infections and chronic infections [48,49]. The formation of biofilm is a multi-step event that occurs through adsorption or adhesion to surfaces of medical implants, the release of extra polymeric substances (EPS), colony formation, and biofilm maturation [48,49,50]. Biofilm formation is driven by quorum sensing (QS), defined as the process of the excretion of small signaling molecules that allow microorganisms to communicate. These small diffusible molecules include acylated homoserine lactones that bind to the promoter of the QS target genes, affecting their expression. Other signaling molecules have been identified in bacteria, including autoinducers, quinolones, indole, pyrones, oligopeptides, and dialkylresorcinols. Furthermore, bacteria apparently do not rely on a single signal molecule, and different QS signaling molecules may act together [51]. Swarming is a complex physiological adaptation process that depends on QS and nutrients availability [27,52]. QS plays a role in regulating biofilm formation [49]; QS enables bacteria to obtain information about cellular density and species composition of their vicinal community and regulate their gene expression profiles accordingly. The signaling molecules of QS have been reported to regulate cellular growth and metabolism, and correlate with biofilm formation and colonization. For example, the signaling molecules can promote the secretion of EPS, an essential step for biofilm formation. As such, QS is important for biofilm formation, enabling pathogenic bacteria to resist environmental conditions, leading to the successful colonization and maintenance of their population, even in the presence of antibiotics [53,54]. CRISPR-CAS, a gene-editing technique, and photodynamic therapy (PDT), are potential therapeutic approaches to mitigate bacterial biofilm infections. The CRISPR-CAS technology has been recently shown to knockdown the luxS gene of QS signaling and the fimbriae-associated gene (fimH), both of which are vital for controlling the biofilm-mediated infections. On the other hand, PDT has significant advantages pertaining to its ability to selectively bind to the membranes of pathogenic cells in biofilms, and accurately deliver light to the affected tissue where the biofilm exists, for the maximal damage of microbes, as well as the minimal damage of the host [49].

#### 2.3.3. Acquired Resistance

Acquired antibiotic resistance mainly occurs due to HGT and bacterial chromosomal DNA mutations. The acquisition of genetic material can be either temporary or permanent [40]. The plasmid-encoded specific efflux pump leads to the enhanced expression and dissemination of outside genetic resistance determinants [42,55,56], and is the most common route for acquiring resistance, while bacteriophage-mediated transmission is considered rare [40]. This genetic exchange occurs through three main mechanisms widely used in nature. Certain bacterial species have a predilection to one or more of the following resistance mechanisms, which include transformation by incorporating naked DNA, transduction that is phage-mediated, and conjugation by cell-to-cell contact [57].

The physiological role of transformation is DNA repair or genetic diversification to enhance adaptability [58], depending on the nutritional status of the bacterium on one hand [59], and on the environmental stressors on the other hand [60]. Transformation played a role in the evolution of antibiotic-resistant strains of *Streptococcus* spp. [58]. *Transduction* may be involved in the evolution of resistance in *Staphylococcus aureus* [61] and many other bacteria at a low frequency ranging between 106 and 109 transductants/plaque-forming unit [62,63,64]. Transformation and transduction mechanisms are difficult to detect outside of laboratory settings, and results related to their role in conferring resistance are unclear [42]. However, some hotspot environments for genetic exchange, such as sewage and wastewater treatment plants, are prime locations for exchange events, because of the high density of bacteria, phages, and plasmids in these settings [65,66].

Plasmid-mediated conjugation is the most prevalent mechanism in spreading resistant genes [67] against different classes of antibiotics. The plasmid can replicate autonomously and transfer genes over long genetic distances to the host [68,69]. Gene transfer by conjugation contributes to the global spread of resistant determinants in the community and the hospital environment [70]. This mechanism occurs in high-density settings, such as the human or animal gut, biofilms, and co-infection conditions [71,72,73]. Conjugation is the most extensively studied mechanism by DNA sequencing and PCR-based approaches [70]. Conjugation can occur as a response to the environmental selection pressure, where some resistance determinants use mobile genetic elements (MGEs), the plasmids and transposons, as vehicles to share valuable genetic information, while others transfer plasmids from chromosomes [57]. Integrons are another highly efficient mechanism for accumulating antimicrobial resistance genes. These are site-specific recombination systems capable of recruiting open reading frames in the form of mobile gene cassettes [42].

Mutations contributing to antibiotic resistance usually occur in three main types of genes: The genes encoding antibiotic targets, the genes encoding their transporters, and the genes encoding regulators that repress the expression of transporters [29]. The average mutation rate was estimated as 1 for every 106 to 109 cell divisions [40]. Bacterial mutations can occur spontaneously without mutation induction due to errors during DNA replication, or through exposure to chemical or physical or biological mutagens [74]. Internally, genetic mutations can also occur by insertion sequences and integrins that move genetic material around [40].

### 2.4. Mechanisms of Antibiotic Resistance

**Figure 1:**
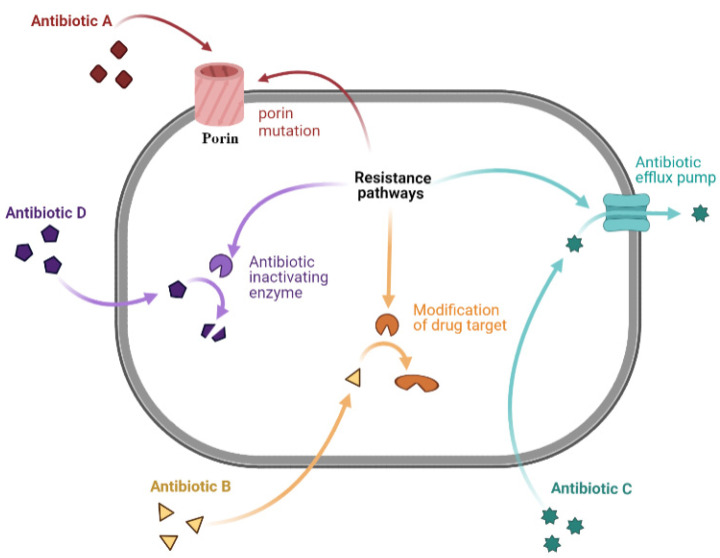
Representation of a bacterial cell, illustrating the common mechanisms of antibacterial resistance. Antibiotic A corresponds to the mechanism of limiting drug uptake; B to the modification of drug target; C to antibiotic efflux; and D to drug inactivation by bacterial enzymes. Figure was prepared using Biorender.com (accessed on 20 January 2022).

Antibiotic resistance mechanisms include limiting antibiotic uptake, modifications of the antibiotic target, antibiotic inactivation, and active antibiotic efflux [40]. GNB can adapt to the four listed mechanisms, and the GPB bacteria make less use of the limiting antibiotic uptake, owing to the lack of a lipopolysaccharide outer membrane [40,75]. The mechanisms of resistance are summarized below and illustrated in Figure 1.

#### 2.4.1. Limiting Drug Uptake

Antibiotics exert their action in clinical practice by reaching intracellular bacterial targets [75]. A decrease in antibiotic permeability of the outer membrane limits antibiotic influx [75]. This mechanism is of interest in GNB due to lipopolysaccharide (LPS), a component of the GNB outer membrane. These changes affect hydrophilic molecules, such as vancomycin and fluoroquinolones, which often use porins as a water-filled diffusion channel to cross these barriers [68,76].

#### 2.4.2. Modification of Drug Target

Chemical alterations of antibiotics occur through the production of enzymes seen in acquired antibiotic resistance in GNB and GPB [77]. However, in GPB, resistance occurs through the modification of antibiotic targets, like penicillin-binding proteins (PBPs). These differences are probably due to differences in the structure of the cell wall/envelope between the two types of bacteria [77]. PBPs are transpeptidases involved in building the bacterial cell wall peptidoglycan. Any modification in their number can affect the number of antibiotics that can bind to the target, and any change in their structure may decrease or inhibit antibiotic binding [40]. Changes in target sites are bacterial mechanisms that consist of target replacement and protection [75].

Resistance mechanisms may be due to: (i) The acquisition of the *van* gene cluster that occurs on MGEs [78] and results in changes in the structure of peptidoglycan precursors, leading to a decline in the binding capacity of vancomycin [40]; (ii) a ribosomal protection mechanism, as in tetracyclines, or targeting the ribosomal subunits via ribosomal mutations, as in aminoglycosides, or a ribosomal subunit change, as in the enzymatic alteration of chloramphenicol, an antibiotic that inhibits protein synthesis by interacting with the peptidyl-transfer center of the 50S ribosomal subunit [79].

Another type of enzymatic alteration seen in macrolides resistance is conferred by a large group of erythromycin ribosomal methylation (*erm*) genes [80]. These, like *van* genes, also occur on MGEs, and they are widespread among both GPB and GNB [81].

#### 2.4.3. Drug Efflux

Drug efflux is an antibiotic resistance mechanism seen in clinical isolates. Genes encoding antibiotic efflux pumps can be either intrinsic or acquired. Many classes of active efflux pump are characterized in both types of bacteria that belong to five families: (1) the ATP-binding cassette family (ABC), (2) the major facilitator superfamily (MFS), (3) the small multidrug resistance family (SMR), (4) the multidrug and toxic compound extrusion family (MATE), and (5) the resistance-nodulation-cell-division family (RND) [82]. This family classification of efflux pumps depends on their composition, nature of substrates, energy source, and number of transmembrane spanning regions. The ABC family is mainly found in GPB, uses ATP as an energy source, and has a limited role in conferring MDR. The MFS superfamily, mostly present in GPB and the RND family, characteristic of GNB, is mostly associated with antibiotic resistance. The SMR family includes the smallest drug efflux proteins known, while the MATE family includes pumps that can resort to the sodium gradient as an energy source [83]. As such, these families demonstrate differences in the structure and uses of energy sources, where only ABC proteins use the ATP source of energy, while the other couple transports substrates to ion gradients [42,75]. One of the most frequently encountered efflux-mediated resistance mechanisms is the one depending on the MFS family that extrudes tetracycline using proton exchange as the source of energy [81].

#### 2.4.4. Drug Inactivation

Drug inactivation occurs via the actual degradation of the drug by hydrolytic enzymes, like β-lactamases and the oxygen-dependent destruction of tetracyclines catalyzed by the enzyme TetX. Drug inactivation occurs by transferring a chemical group to the drug, such as acetyl, phosphoryl, and adenyl groups [40]. A frequently encountered example is seen in cases of resistance to aminoglycosides, where different types of aminoglycoside modifying enzymes (AMEs), which include both N-acetyl transferases (AAC) and O-phosphotransferases (APH), have been found in both GPB and GNB [69].

### 2.5. The Spread of AMR: The Known and the Unknown

Much is known regarding the spread of AMR in clinical practice, but there are also a number of unknowns, particularly from a One Health perspective. Poor infection prevention and control (IPC) measures are well known to result in the spread of AMR [84]. However, the ability to conduct studies to address the disparities which prevent effective IPC programs, especially in low- and middle-income countries (LMICs), has been affected by a number of factors. These include limited or restricted resources, lack of training in IPC, lack of computer infrastructure to allow for appropriate surveillance, and multiple competing political agendas [85]. From the perspective of animal health, poor IPC at the level of animal health clinics have also been shown to cause environmental contamination, with resultant colonization transmitting from animals to humans in that environment [86].

The presence of resistance genes on MGEs has accelerated the spread of AMR from one organism to another, further expediting global spread. A recent systematic phylogenetic analysis conducted by Wang et al. demonstrated that all currently circulating *mcr-1* elements globally descend from the mobilization of a single gene by a transposon around the year 2006, and points to its possible origin in Chinese livestock [87]. The environmental and impending climate crisis has also been regarded as a reservoir for the spread of AMR. For instance, it is postulated that *Candida auris* may have emerged in the environment first, and subsequently spread to humans as a result of thermal adaptation due to climate change [88]. This theory was supported by the subsequent isolation of *C. auris* from the coastal wetlands of the Andaman Islands, India [89]. Similarly, bacterial pathogens harboring carbapenemase genes have been found in a specified geographic area in both humans and the environment in South Africa, suggesting possible spread from one reservoir to the other [90].

**Figure 2:**
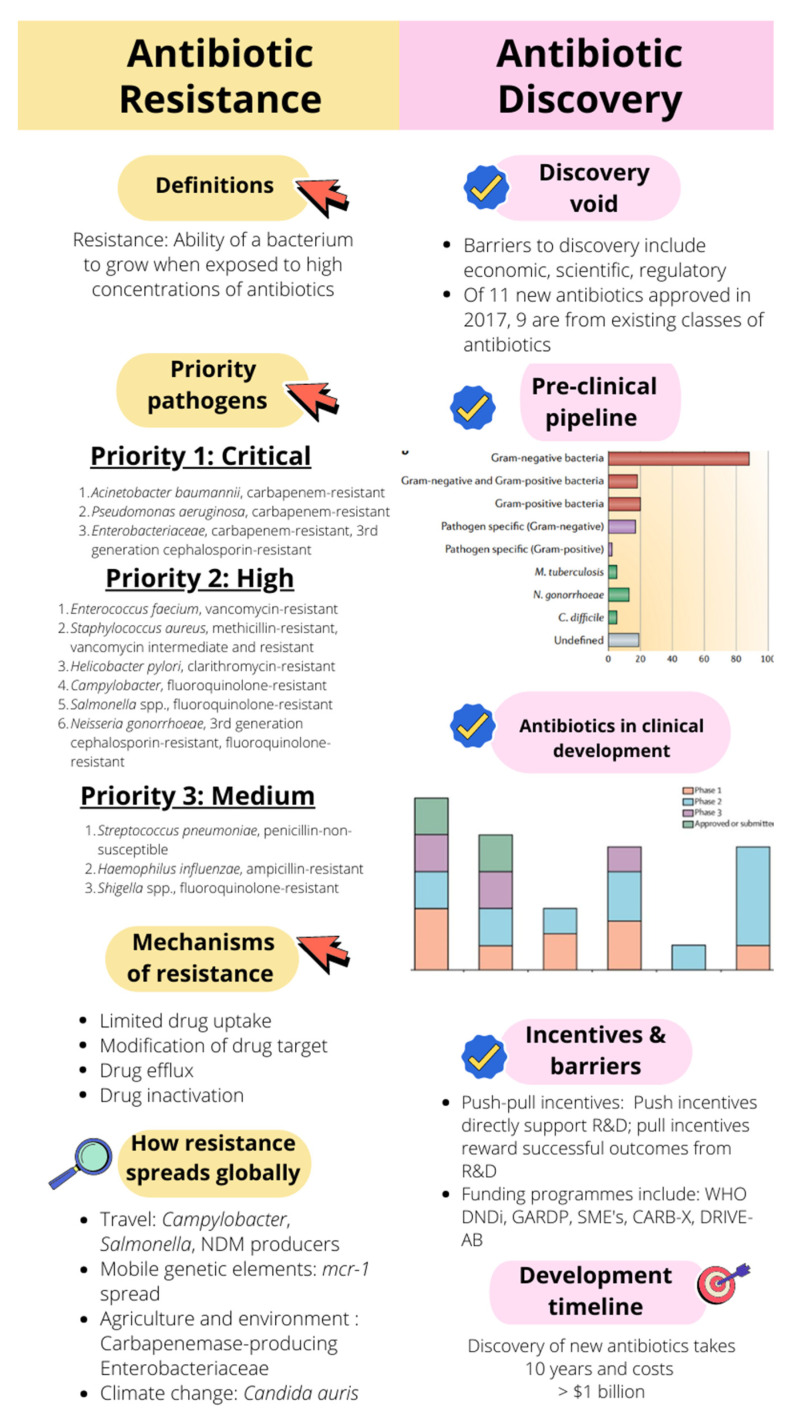
The basic concepts of antibiotic resistance (the chase) and antibiotic discovery (the race) discussed in this article. Pre-clinical pipeline graph: [13]. Antibiotics in clinical development: [14]. WHO DNDi: Health Organization Drugs for Neglected Diseases Initiative; GARDP: Global Antibiotic Research and Development Partnership; SMEs: Small and Medium-Sized Enterprises; CARB-X: Combating Antibiotic-Resistant Bacteria Biopharmaceutical Accelerator; DRIVE-AB: Driving reinvestment in research and development for antibiotics and advocating their responsible use.

Yet, there are still a myriad of unanswered questions related to the spread of AMR. We are currently not able to quantify the relationship between agricultural antimicrobial use and the development of AMR, or how much agricultural antimicrobial use contributes to the risk of AMR in a clinical setting. The identification of AMR selection hotspots is also essential to prevent transmission to the clinical setting and prevent outbreaks. How environmental AMR contamination or emergence results in human and animal infection requires more detailed analysis. There is currently no single database which is able to collate information on antimicrobial use and antimicrobial development and spread [91]. However, a sigmoid model used against the surveillance databases of several European countries has been able to describe the spread of AMR more accurately than linear models, taking into account country-specific dynamics [92]. The ability to predict AMR is becoming of increasing importance in containing its further spread. To this end, whole genome sequencing coupled with machine learning models have been used to predict resistance to GPB and GNB in a clinical setting [93,94]. Figure 2 illustrates the key components of antibiotic resistance (the chase) and antibiotic discovery (the race), with a summary of some concepts discussed in this article.

### 2.6. Drivers of Antimicrobial Resistance

The global drivers of AMR are complex, interdependent [95,96,97,98,99] from a One Health perspective, and inter-connected through international trade and travel [100], as well as migrant carriers; namely, refugee and asylum seekers [101]. The WHO reports that global expenditures on health may differ tremendously between high-income countries (HICs) and low-income countries (LICs), where health and subsequently AMR may not be a high national priority [102,103,104,105]. These differences may be related to the lack of awareness and education about AMR, poverty, hunger, poor sanitation, overcrowdedness, urbanization, loose regulations, and health inequity issues. Considering health as a priority is a political decision, and this applies to AMR. Multiple cultural, economic, and political dynamics may shape individual behavior and response to crisis [106]. The level of education may influence the individual’s accountability, but may not necessarily lead to a more rational antibiotic use [107]. Health systems governance and leadership, and the magnitude of the government’s engagement to secure social welfare, have major impacts on the systemic approach to mitigate AMR. The indiscriminate use of antibiotics for prophylactic, metaphylactic, and treatment objectives [106], and the increase in the global consumption of antimicrobials in food/animal production, have intensified the use of antibiotics to meet global demands [97,104,108]. The balance between doubling agriculture production and decreasing the uses of antimicrobial agents while securing food safety seems impossible. The global problems of hygiene, sanitation, water contamination, sewage, manure run-offs, and hazardous waste are additional challenges that influence the wide spread of AMR [95,96]. In some countries, slaughter and butcheries waste is released into the environment [109,110,111], untreated animal waste serves as fertilizer [102], and surface water is a shared resource between humans and animals [112]. Poorly managed hazardous waste may reach groundwater, drinking water, soils, food crops, and sediments [113,114]. The latter can have detrimental environmental effects which affect wildlife and generate antibiotic-resistant bacteria, causing widespread illnesses in the community [113]. Heavy metals and biocides have been associated with the co-selection and dissemination of resistance in environmental bacteria [115]. Heavy metals are found in the soil from agriculture and mining origins, and as trace elements of the antimicrobial growth promoters (AGPs) used in livestock production. Biocides are frequently encountered within the food and agriculture industry, intended for use as animal food preservatives, and within healthcare settings, as disinfectants and decontaminants. These are associated with the dissemination of AMR in environmental bacteria due to the decreased susceptibility of bacteria in the soil [116]. Biocides and antimicrobial agents can share common target sites [117] and can be located closely together in mobile units [118]. Many genes encoding resistance to these agents have been linked to antibiotic resistance genes in single genetic elements, leading to cross- and co-selection for antibiotic resistance [117,119].

Limited access to resources such as broad-spectrum antibiotics [120] may lead to the underutilization of antibiotics, or drive one unknowingly to the choice of counterfeit medications, due to their lower price [121,122,123,124]. Other drivers may be related to the direct access to antibiotics through illegal online vendors and over-the-counter dispensing [125,126,127], owing to the loose rules and regulations in some countries [128]. Studies indicate that nearly half of the global antibiotic consumption is not justified [129]. Physician over-prescription is well-documented, due to multiple causes that may be ethical, and sometimes unethical [130]. The pharmaceutical companies’ tendencies to overproduce and maximize the sales of antibiotics before the expiry of drug patency [131,132], and the lack of incentives to invest in the research and development of new agents, are major contributors to the scarcity of resources [131]. In brief, AMR is a crisis of many hands, and it mandates a context-specific systemic approach to multiple disciplines using different models, theories, and concepts [133].

### 2.7. Priority Pathogens 

The WHO global priority pathogens list published in 2017 emphasized the antibiotics urgently needed for research and development [134]. This list highlighted the threat of Gram-negative multi-drug-resistant (MDR) bacteria. The WHO grouped the global pathogens into three categories: The critical priority group is considered a threat in healthcare settings, predominantly among immunocompromised patients requiring ventilators and the insertion of blood catheters. This group includes antibiotic-resistant pathogens with a majority of GNB. The other groups labeled as high and antibiotic medium priority consist of Gram-positive- and Gram-negative-resistant bacteria associated with commonly seen infections. In 2019, the Center for Disease Control and Prevention (CDC) released an updated list of resistant pathogens grouped into urgent, serious, and concerning, in addition to the WATCH list (Table 1) [135]. The WHO and CDC lists are an urgent call for more targeted research and development for new antibiotics, taking into consideration the current incidence of resistance, documenting the economic and clinical impact of pathogens, in addition to the transmissibility of the bacteria, the availability of effective antibiotics, and the barriers to prevention. Another factor to be considered is the international spread of AMR through travel and import [135]. Meanwhile, the global spread of antibiotic-resistant bacteria continues to outpace innovative antibiotics discovery [136].

## 3. History of Antibiotics

### 3.1. The Pre-Antibiotic Era

Exposure to antibiotics was documented long before the advent of modern medicine [137,138]. Traces of tetracyclines were discovered in human skeletons in Nubia, in ancient Egypt [139,140,141,142]. A study of the femoral midshaft of the samples of a Nubian excavated skeleton showed potential evidence of the exposure to tetracycline in the diet during this ancient period. The topical application of moldy bread to treat skin infections and the speed healing of wounds and burns have also been documented in ancient Egypt, Rome, Greece, Serbia, and China [137,143]. Some herbs used as remedies in traditional Chinese medicine showed some antimicrobial activity postulated to be a contributory factor for the accumulation of antibiotic resistance genes in human populations over the years [144]. Pyocyanase was the first antibiotic used in hospitals to treat human infections [143]. This drug was prepared by the two German physicians, Emmerich and Löw, who discovered that *Pseudomonas aeruginosa* isolated from injured patients’ bandages inhibited the growth of several pathogenic bacteria, such as those causing anthrax, cholera, typhoid, and diphtheria [44,145]. Emmerich and Löw created, in the 1890s, a medication based on extracts of *P. aeruginosa*, and called it Pyocyanase. They obtained mixed results with this drug, which exerted a toxic effect in humans due to phenazines [143]. Further research demonstrated that some compounds derived from *Pseudomonas* genus, such as alkyl quinolones, appeared to be the quorum-sensing molecules that display potent antimicrobial activities [146,147,148,149]. In 1877, the concept of a microbe inhibiting another microbe started with Louis Pasteur and his colleague Jules François Joubert, who discovered that co-cultivated aerobic bacteria inhibited the growth of *Bacillus anthracis* [137].

### 3.2. The Antibiotic Era

Three antimicrobials marked the beginning of the antibiotic era: salvarsan, prontosil, and penicillin [44]. In 1909, salvarsan was discovered by Paul Ehrlich [143], an influential researcher and a cofounding pioneer of the fields of chemotherapy, pharmacology, hematology, and immunology. His work contributed to the foundation of translational medicine [150]. Ehrlich was first interested in developing stains intended for the histological examination of tissues when he noted that some strains were toxic for some bacteria [143], and started the concept of the magic bullet, known nowadays as the targeted therapy [150]. This idea led to both the development of salvarsan, the first modern antimicrobial agent that proved its effectiveness for the treatment of syphilis, and the initiation of large-scale screening programs [143]. In 1914, neoarspheniamine became known as neosalvarsan, a less toxic product, with the same indication as, and better effectiveness than, the former drug marketed by Hoechst, until it was replaced in the 1940s by penicillin [143,150]. This systematic approach introduced by Ehrlich became the cornerstone of drug screening strategies in the pharmaceutical industry, which resulted in the identification of thousands of antimicrobial drugs [44]. German scientists at Bayer followed Ehrlich’s path and examined the antibacterial effects of dyes. In 1908, Bayer chemists synthesized sulfanilamide, and combined it with a dye to produce prontosil. The drug effectively treated streptococcal infections in mice in 1932, in research conducted by Gerhard Domagk [151]. In 1935, researchers found that adding the dye has no advantage, and sulphonamide emerged as an antimicrobial treatment [152].

The discovery of penicillin is linked to Sir Alexander Fleming (1881–1955), a Scottish physician. Nevertheless, the antibacterial properties of molds were described much earlier, in 1870, by Sir John Scott Burdon-Sanderson (1828–1905), and the year after, by Joseph Lister (1827–1912), who demonstrated that ‘*Penicillium Glaucium*’ had an antibacterial effect on human tissues, and in 1875, Dr. John Tyndall (1820–1893) presented his research findings on *Penicillium notatum* to the Royal Society [143]. In 1897, Ernest Duchene noted that some molds kill bacteria. He discovered the inhibitory effect of *Penicillium glaucum* nearly 30 years before Sir Fleming [137]. In 1928, Fleming discovered that the mold *P. notatum* inhibited *S. aureus* in a plate culture [44,143]. He discovered lysozyme in 1922, an enzyme with weak antibacterial activity [153]. Fleming tried for 12 years to raise the interest of chemists in the purification and stabilization of the drug, but eventually abandoned the idea in 1940. The same year, Howard Florey, a pharmacologist and pathologist, and Ernst Chain, a biochemist working in Oxford University, published a paper describing the protocol of penicillin purification that eventually led to the production and marketing of the antibiotic in 1945 [154].

**Figure 3:**
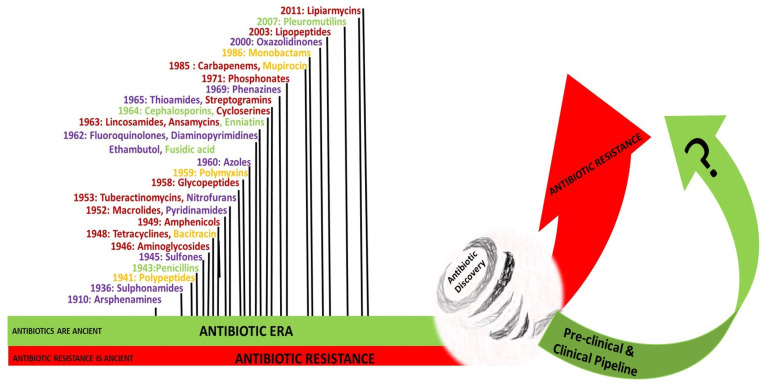
The chase and the race between antibiotics and antimicrobial resistance. The classes of antibiotics and dates of clinical introduction into the market are shown. Purple color: Synthetic antibiotics; gold color: antibiotics from other bacteria; green color: antibiotics from fungi; red color: antibiotics from actinomycetes [154].

The *golden era* of antibiotics discovery was between the 1940s and 1970s [137]. In 1939, René Dubos discovered Gramicidin, the first clinically tested antibiotic [155]. Dubos’ early experiments were based on two principles; the first was the principle of “antibiosis”, defined in 1889 by Jean-Paul Vuillemin as “one living organism kills another to ensure its own existence”, and considered the soil as a “self-purifying environment that could supply an agent to destroy disease-causing bacteria” [137,155,156]. Dubos and biochemist Rollin Hotchkiss examined and analyzed the chemical nature of the antibacterial substance produced by *Bacillus brevis*, and showed that the active substance tyrothricin contained tyrocidine, a lysin that attacked the membranes of both GPB and GNB, and gramicidin, a bacteriostatic agent that selectively inhibits GPB [155]. The systematic approach of screening the antimicrobial activity of soil bacteria, particularly *Streptomyces*, was first undertaken in 1940 by Selman Waksman, who developed multiple culture techniques and strategies known as the “Waksman platform” [137]. This platform inspired the pharmaceutical industry and led to major antimicrobial discoveries between the 1940s and 1970s, starting with streptomycin, which was isolated in 1944 from *Streptomyces griseus* [137]. A program initiated by Eli Lilly and company in the 1950s led to the discovery of vancomycin in 1952, which was extracted from a soil sample sent from a missionary in Borneo that grew *Streptomyces orientalis*. Vancomycin became available for patient use in 1958 [157]. The genus *Streptomyces* is considered the source of nearly one-half of antimicrobial agents currently available in the market for the treatment of infectious diseases in humans [137]. The pharmaceutical industry abandoned the Waksman platform, and switched to the in vitro synthesis of new molecules [158]. The new antibiotics were either a modification or an improvement of known molecules. Figure 3 illustrates the different classes of antibiotics and clinical availability dates, with a highlight on the chase between resistance acting like a tornado devastating and damaging achievements in antibiotic discovery, and the race to develop new antibiotics expected to overcome the escalating spread of AMR.

## 4. Lists of Critically Important Antibiotics for Human Medicine

The WHO List of Critically Important Antimicrobials for Human Medicine (WHO CIA List) was first developed in 2005, and later updated six times according to expert recommendations during meetings organized by the Food and Agriculture Organization of the United Nations (FAO), the World Organisation for Animal Health (OIE), and WHO [159]. Antimicrobials are categorized based on two criteria: (1) The availability of the antimicrobial agents (sole or one of the limited available therapies to treat serious bacterial infections in human medicine), and (2) their uses in high frequencies in human medicine or risk groups for the treatment of infections caused by resistant bacteria or genes transmitted to humans from non-human sources [159]. Antibiotics that meet both criteria are the highest priority, critically important antimicrobials (CIA). This list includes third, fourth, and fifth generation cephalosporins, glycopeptides, macrolides, ketolides, polymyxins, and quinolones. Antibiotics are categorized as highly important if one of the criteria is met, such as first and second generation cephalosporins, lincosamides, penicillins (aminopenicillins), penicillins (anti-staphylococcal), sulfonamides, and tetracyclines. If neither criteria are applied, the antibiotic is labeled as important, such as cyclic polypeptides and nitrofuran [159].

## 5. The Antibiotic Pipeline and the Discovery Void

Diversity and innovation are the mainstays to cope with the rapid evolution of antimicrobial resistance [160]. The discovery and development of antibiotics must address the epidemiology and evolution of resistance towards old and novel antibiotics, and the gaps in the coverage of priority pathogens spread in different geographic areas [13,161,162]. The WHO considers that there is “no time to wait” to secure a future free from drug-resistant bacteria, and points to the scarcity of new antibiotic leads that may jeopardize the global actions undertaken to mitigate AMR [163]. The WHO 2020 report on antibacterial agents in clinical and preclinical development shows that the clinical pipeline and the newly approved antibiotics are not enough to mitigate the emergence and spread of AMR [11]. The WHO sets four criteria for innovativeness which include (1) new target, (2) new mode of action, (3) new class, and (4) the absence of known cross-resistance. Ideally, the optimal lead compound should present no cross-resistance, a novel mechanism of action, a favorable pharmacokinetic profile, and a proven efficacy and safety profile [14,143,164]. The limited number of antibiotics approved by the US Food and Drug Administration (FDA) and European Medicines Agency (EMA) since July 2017 have limited the clinical benefit over pre-existing antibiotic classes, and offer a short-term activity against selected bacterial species, owing to the documented selection pressure against pre-existing antibiotics [11]. Of the eleven new antibiotics, nine are derived from existing new classes (80%) with documented resistance mechanisms, and an anticipated rapid emergence of resistance. Five of the newly approved drugs target carbapenem-resistant Enterobacteciae (CRE). Only one has the ability to penetrate the outer membrane of GNB and accumulates in the periplasmic space. This agent is active against three critical GNB, which include CRE, carbapenem-resistant *Acinetobacter baumannii* (CRAB), and *P. aeruginosa* (CRPA), and has activity against a variety of β-lactamases, including ESBL and AmpC. The two new agents that met at least one WHO criterion for innovativeness are active against *K. pneumoniae* carbapenemase (KPC)-producing carbapenem-resistant Enterobacteriaciae (CRE). The other agent is intended for topical administration in human medicine, and for systemic use in veterinary medicine. One agent was approved as a treatment of extremely drug resistant tuberculosis (XDR-TB) and drug-intolerant or non-responsive MDR-TB. The quantitative and qualitative analysis of the pipeline indicates that few new antibiotics approvals are expected soon, and the current pipeline is still insufficient to tackle the challenging spread of AMR. Most new agents in the clinical pipeline targeting GNB and GPB lack innovation [14,164]. Very few leads target the critically important Gram-negative priority pathogens that are causing a substantial global concern due to the rapid emergence and spread of the critical MDR and XDR GNB species [14,143,165]. The challenges of antibiotic discovery targeting GNB remain in the difficulty overcoming the GNB cell wall, which includes two lipid bilayer membranes, porins, and efflux pumps [14,166]. An analysis of the current clinical antibacterial pipeline (Phase 1–3) shows that it is still dominated by β-lactam and β-lactamase inhibitor (BLI) combinations (40%), with a major gap in activity that targets metallo-β-lactamase (MBL) producers. Other agents include tetracyclines, aminoglycosides, topoisomerase inhibitors, oxazolidinones, macrolides, ketolides, polymyxin, antibiotic hybrids, and *FabI inhibitor* (FabI is a NADH-dependent enoyl acyl carrier protein reductase, encoded by *fabI*), and filamenting temperature-sensitive Z (FtsZ) inhibitors [11]. As of September 2020, the clinical pipeline includes 68 new chemical entities, among which 18 are thought to fill the four innovativeness criteria. There are a total of 41 antibiotics and combinations with novel entities (chemical entity, biological entity, new substance, and new molecular entity), and 27 non-traditional antibacterial agents. Twenty-four antibiotics target the WHO priority pathogens, twelve target *M. tuberculosis*, and five *C. difficile.* [11]. Nineteen non-traditional antibacterials are targeting the priority pathogens, and eight target *C difficile.* Of the 27 non-traditional antibacterial agents in the clinical antibacterial pipeline, 18 are active against GPB (*S. aureus* and *C. difficile*), and 7 against GNB (*P. aeruginosa* and *E. coli*, and *Campylobacter jejuni*). Two have broad-spectrum activity that targets GNB and GPB. The diversity of these agents is related to the non-traditional approaches that include antibodies (9 agents), bacteriophages (4 agents), and phage-derived enzymes, microbiome-modulating agents (8 agents), immunomodulating agents (2 agents), and miscellaneous agents, which include anti-virulence agents (4 agents) [11]. A summary of antibacterial products in current phases of development is shown below in Table 2.

The preclinical pipeline is described as dynamic, and includes a broad range of drug development projects that show innovativeness, and use variable approaches to target the WHO bacterial priority pathogens list, *M. tuberculosis* and *C. difficile*. The 2020 WHO database captures a total of 292 antibacterial agents progressed by commercial entities (87.6%), and non-commercial entities like academic institutions (10.5%), and foundations (1.8%). The developers are widely geographically spread, primarily in the European region (44%), followed by the Americas (39.5%), the Western Pacific region (12.3%), and the Southeast Asia region (3.7%). The preclinical agents under development are direct-acting small molecules (39.4%), non-traditional products (34.6%), vaccines (16.1%), and adjuvant antimicrobial peptides (9.9%). The antibacterial mode of action target cell wall synthesis (13.7%), cell membrane (21.1%), DNA replication (5.5%), protein synthesis (9.6%), RNA synthesis (1.7%), cell metabolism (2.1%), immunomodulation (19.2%), anti-virulence (7.5%), others (8.9%), and undisclosed modes of action (10.6%) [11]. Although the pre-clinical pipeline offers diversity and innovation, there is a potential that it may not progress into beneficial clinical effect, due to a high attrition rate, high capital risk, negative return on investment, and multiple other scientific and translational challenges [12,13,166,167].

## 6. Methods of Antibiotic Discovery

**Figure 4:**
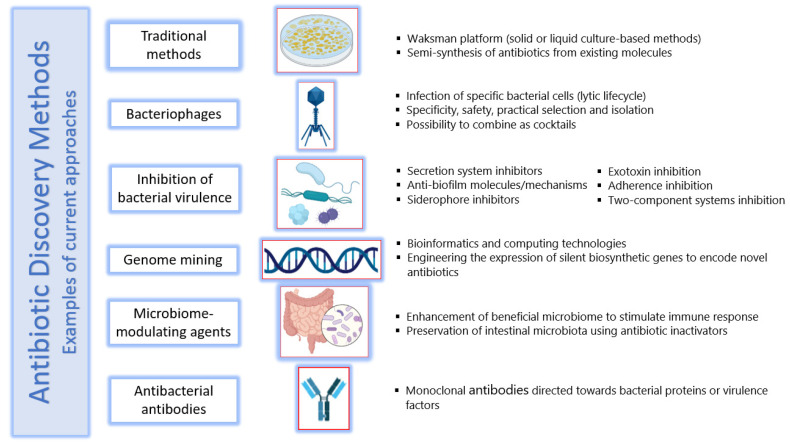
Concise representation of some antibiotic discovery methods and new approaches to design therapies that circumvent antimicrobial resistance. Photos were prepared using Biorender.com (accessed on 20 January 2022).

Following the mid-1950s, a gradual decline in antibiotic discovery and the evolution of drug-resistant pathogens have led to the current antimicrobial resistance crisis. However, according to available evidence, the future of antibiotic discovery looks positive, as new technologies such as genome mining and editing are deployed to discover new natural products with diverse bioactivities [168]. Beyond conventional antibiotics, some interesting therapeutic alternatives are being discovered and studied, including bacteriophages, antivirulence compounds, probiotics, vaccines, immune stimulation, antimicrobial peptides, antibiofilm therapies, and antibodies, among others. Despite the fact that some of these alternatives reached clinical trials, it is estimated that across the next decade or so, over GBP 1.5 billion will be needed to further test and develop them before their clinical influence is sensed [169]. The current status of antibiotic development should be upgraded to align with the race of multi-resistant pathogens; it ranges from traditional techniques to innovative approaches, a bouquet of which is presented below. A representation of some of the methods is shown in Figure 4.

### 6.1. Traditional Methods

Since 1937, the Waksman platform has been well known as a culture-based method for antibiotic discovery. Provoked by the remarkable successes at the beginning of the 20th century, Selman Waksman noticed that complex soil bacteria and actinomycetes inhibited the growth of other bacteria, and acknowledged such competitive growth, which could become the conceptual basis of a screening method for antibiotic-producing organisms [170]. These methods are based on the inhibition of a test strain over a closely cultivated indicator strain. The test strain is the strain suspected to produce an antimicrobial, targeting the strain used as an indicator. Several techniques within such culture-based methods exist to detect antimicrobial activity, either in solid or liquid culture [137].

Following the application of the Waksman platform, antibacterial semi-synthesis by the modification of existing scaffolds or molecular backbones came into action. This provided chemical stability and the reduction of undesirable side effects, among other features that are crucial in marketing antibiotics. For example, the semi-synthesis from penicillin expanded this drug from a single entity to a range of semi-synthetic derivatives, constituting an entire class of beta-lactam antibiotics [171]. The rate at which derivatives with improved properties can be synthetized maintained control against infectious diseases, a key characteristic of semi-synthesis [170]. However, resistance to these semi-synthetic antimicrobials has been rapidly increasing, and the race in their high rate of prescription highlights the importance of chasing them by continuously developing novel derivatives.

### 6.2. Bacteriophages

The use of bacteriophages, viruses that infect bacteria, as agents to treat bacterial infections began two decades before the first clinical use of an antibiotic. However, the introduction of broad-spectrum antibiotics in the 1940s rapidly concealed and banished the development of phage therapeutics [171]. Bacteriophages infect their specific bacterial hosts and, in the lytic lifecycle, takeover the machinery of the host cell to replicate and ultimately destroy the host, producing a progeny of themselves and killing the host. Being biologically abundant and diverse, phages provide a ready resource for a variety of purposes, including not only anti-bacterial therapy, but also decontamination, infection control, detection, and diagnosis [172]. Phages offer potential advantages as antibacterial therapeutics, including effectiveness against MDR pathogens; having high specificity and, thus, little to no effect on normal microflora; targeting precisely the tissues needed to eliminate pathogen cells; possessing ease of selection and isolation; encoding enzymes that degrade the biofilms that can be associated with difficult infections, hence providing access for other antimicrobials to surmount this barrier; displaying safety and low immunogenicity; and being feasible to combine in cocktails to address a diversity of pathogens [173].

A sample study on phages was conducted by Hua and Colleagues, who investigated phages against MDR *A. baumannii* clinical isolates resistant to ciprofloxacin, cefepime, ceftazidime, and piperacillin-tazobactam [174]. The study obtained thirty bacteriophages, and four of them were described: phage SH-Ab 15599, SH-Ab 15708, SH-Ab 15497, and SH-Ab 15519. The phage cocktail containing all four phages was effective against 88% of *A*. *baumannii* isolates, and for one phage, SH-Ab 15519, an enzyme was suggested as the cause of bacterial exopolysaccharide degradation, and was characterized by high absorption (90% within 10 min), and stability from pH 5 to 12, and temperatures from 4 to 50 °C. Recently, a controlled clinical trial using bacteriophages in adult males with complicated urinary tract infection demonstrated the normalization of urine, with a favorable safety profile [175].

After the rekindling of bacteriophages, several questions certainly remain around their ability for horizontal gene transfer, the interaction dynamics with the human microbiome, and the different findings on their immunomodulatory effects; all these need to be answered prior to their application for therapy. However, their integration in antimicrobial research is indeed promising, and an outlook into the future of these smallest ever creatures may prove them to be greatly useful.

### 6.3. Inhibition of Bacterial Virulence

In its broad sense, virulence can be defined as the relative capacity of a microorganism to cause damage in a host, and results from complex pathogen–host interactions. A virulence factor is defined as any genetic attribute that increases the chance to cause disease in a host [176]. Examples of important bacterial virulence factors include enzymes, toxins, exopolysaccharides, metal-acquisition systems, and two-component signaling systems, as well as surface structures, including lipopolysaccharides, capsules, glyco- and lipoproteins. One compelling intervention to respond to the current antimicrobial resistance and anticipate evolving resistance mechanisms to antibiotic therapy is the development of antivirulence strategies, by which only virulence, but not the survival/fitness traits of a bacterial resistant pathogen, is targeted [177]. Another intervention is reversing AMR by targeting chromosomal non-essential genes that are not responsible for acquired resistance, but are essential for resistant bacteria under the therapeutic concentrations of antimicrobials. This approach suggests that the secondary resistome is a potential target for developing antimicrobial “helper” drugs that restore the efficacy of existing antimicrobials, and is in a way, affecting the virulence of resistant pathogens [178,179,180,181].

Several approaches to circumvent virulence are studied; for example, Gram-negative bacteria have evolved a broad range of secretion systems to transport small molecules, proteins, and DNA into the extracellular space, or into target cells. A remarkable array of such sophisticated nanomachines is used to deliver various virulence factors across the bacterial cell envelope [182]. These dedicated secretion systems are numbered type I through type VI, with each system transporting a specific subset of proteins. All these systems rely on the so-called β-barrel channels that commonly form a ring in the bacterial cell outer membrane, but otherwise display a reasonable amount of diversity in their structures and mechanistic functions.

The secretion systems most innately related to pathogenesis are the type III secretion system (T3SS) and type IV secretion system (T4SS). Both are highly conserved in structures among pathogens, and span the bacterial inner and outer membranes to connect to the host cell. Here, they form a pore in the host cell membrane, enabling the secretion of virulence proteins directly into the host cytosol, interfering with normal functions to help in bacterial persistence [177,183]. These secretion systems appear to be good candidates for targeting bacteria: The structures and functions of the secretion systems, as well as their synthesis and assembly, show a high conservation between the different strains of bacteria. Proteins forming the secretion systems are exposed on the bacterial cell surface, making them accessible. If inhibitors of these systems are found, these inhibitors can target not only a single, but rather several different pathogens at once. Moreover, as only pathogenic bacteria express these types of secretion systems, non-pathogenic species or normal microbiota will not be targeted. Additionally, as secretion system inhibition does not influence the overall survival of the bacterium, the selective pressure to develop resistance is low [177].

One of the very first type III secretion system inhibitors was an antimicrobial glycolipid named caminoside A, derived from extracts of the marine sponge *Caminus sphaeroconia*, and was effective against *Escherichia coli* [184]. Moreover, guadinomines are *Streptomyces*-produced natural compounds with a strong activity against the III secretion system of enteropathogenic *E. coli*. Guadinomines A and B showed potent inhibition, while guadinomine D showed moderate activity [185]. Recent work has demonstrated that the polyketide natural product Aurodox, obtained from *Streptomyces goldiniensis*, downregulates the expression of the type III secretion systems of enteropathogenic and enterohemorrhagic *E. coli*, and inhibits the expression at the transcriptional level by repressing the master regulator of the secretion system, *ler*. Unlike some traditional antibiotics, Aurodox does not induce the expression of RecA, which is essential for the production of Shiga toxin, making it a promising antivirulence therapy for the treatment of these infections [186].

Another strategy to circumvent virulence is biofilm resolution. A biofilm is a three-dimensional community of bacteria with complex architecture that lives on surfaces, and is encapsulated within an extracellular polymeric matrix of bacterial origin, consisting of a network of hydrated polysaccharides, proteins, and DNA [187]. The structured groups of different bacterial species in a biofilm give propensity for the development of chronic and recurrent infections, which reoccur in approximately 65–80% of cases. Bacteria associated with the biofilm are highly resistant to antibiotics [188]. The mechanical shelter produced by biofilms on bacterial cells makes them impervious to toxic effects of antibiotics, impedes antibiotic penetration, alters bacterial metabolism, increases resistance to stress from UV, acidity, metal toxicity, host immune clearance, and phagocytosis. An additional disadvantage of biofilm formation in terms of infection treatment is the occurrence of processes that lead to the acquisition of inheritable resistance traits, such as horizontal gene transfer and mutation [189]. Therefore, it is important to design or screen anti-biofilm molecules or tactics that can minimize or eradicate biofilm-related infections [190].

One possible targeting strategy for biofilms is biofilm dispersal, which involves the degradation of the extracellular matrix, with the aim of promoting biofilm self-disassembly. This approach assumes that dispersed bacteria return to an active state analogous to their planktonic phenotype, rendering them more susceptible to conventional antibiotics [191]. In this regard, the intracellular secondary messenger nucleotide c-di-GMP plays a basic role of the biofilm maturation of both Gram-positive and Gram-negative bacteria, where increased levels promote biofilm formation, and reduced levels promote disassembly [192]. The modulation of c-di-GMP levels is possible using nitric oxide (NO), which was first shown to regulate c-di-GMP levels and mediate biofilm dispersal in *P. aeruginosa* at low concentrations [193]. However, the use of gaseous NO presents clinical challenges, owing to cytotoxicity from systemic exposure, and lack of specificity in targeting biofilm infections, as well as cost. Additionally, as NO is labile, the optimal concentration for biofilm dispersal is problematic to measure [191]. To facilitate use, the encapsulation of a nitric oxide donor and molsidomine within a hydrogel composed of stable, biocompatible, cellulose nanocrystals was synergistic in dispersing well-established biofilms of Salmonella enterica [194]. Recently, a chitosan-based nitric oxide-releasing dressing showed anti-biofilm and in vivo healing activities in methicillin-resistant *S. aureus* biofilm-infected wounds, with evidence of faster biofilm dispersal, wound size reduction, epithelialization rates, and collagen deposition [195].

For most organisms, iron is an essential trace element used as a cofactor for enzymes involved in fundamental cellular processes [196]. A common way for bacteria to acquire this nutrient is by the secretion of siderophores, small-molecule secondary metabolites that scavenge iron from environmental sources, create soluble Fe^3+^ complexes, and deliver it to bacterial cells via specific receptors [197]. During infection, humans sequester metal ions such as iron in storage proteins to raise the strain for the pathogen scavenging of iron. The ability of a pathogen to acquire iron can determine the course of an infection. The concentration of free iron in the human blood can be as low as 10^−24^ M, while bacteria generally need a higher level of almost 10^−6^ M of iron to survive [198]. The production of siderophores as virulence factors has been validated for many pathogens, including *M. tuberculosis*, *B. anthracis*, *K. pneumoniae*, methicillin resistant *S. aureus* (MRSA), *P. aeruginosa*, and multi-drug-resistant *A. baumanni* [199]. Baulamycin A is a polyketide siderophore biosynthesis inhibitor produced by the soil bacterium *Streptomyces tempisquensis* [199]. Baulamycin A is a reversible competitive inhibitor of siderophore synthetase (NIS) enzymes from *S. aureus* (SbnE) and *B. anthracis* (AsbA) involved in the biosynthesis of staphyloferrin and petrobactin siderophores, respectively. It is capable of stopping siderophore production in liquid cultures of *S. aureus* and *B. anthracis* [200]. The discovery of baulomycin A confirms that natural product extracts can produce lead compounds that are capable of crossing bacterial membranes and hindering siderophore synthesis in a target-specific fashion.

Exotoxins are single or oligomeric proteins produced and released by various bacteria during growth inside the host. Sometimes they cause specific diseases, including diphtheria, anthrax, botulism, and tetanus; some other times, they can act more systemically, attacking many types of cells and tissues, modifying their properties, and causing enormous tissue death and cellular destruction [201,202]. In general, the deletion of exotoxin gene(s) deprives bacteria of basic virulence traits without harming their overall biological fitness. This makes exotoxins ideal targets for new inhibitors, and multiple approaches to prevent the toxin-mediated damage of the host are being investigated [177].

In 2006, a study of high-throughput screen lead compounds led to the discovery of a potent and selective anthrax lethal factor (LF) inhibitor ((2R)-2-[(4-fluoro-3-methylphenyl) sulfonylamino]-N-hydroxy-2-(tetrahydro-2H-pyran-4-yl)acetamide), which is a sulfonamide derivative [203]. The LF, a zinc-metalloprotease, is an anthrax exotoxin critical for infection, and it disrupts host signaling pathways; it is also suggested to contribute to mortality from anthrax infection [204]. The discovered LF inhibitor was found effective in vivo in several animal model studies [177,203]. Later, LF was considered a prime target for inhibitor development to produce anthrax therapeutics. The derivatization of analogs with zinc-binding groups has produced potent and specific LF inhibitors, and the X-ray crystallography of LF inhibitor complexes has provided knowledge about properties required for high affinity binding [205]. For example, a peptide hydroxamate LF inhibitor with high binding potency was developed by Li et al., and had better interaction with LF [206]. They suggested that the sequence optimization of LF inhibitors via the incorporation of preferred amino acids can improve potency. Because the hydroxamate group is susceptible to hydrolysis upon prolonged incubation with LF, converting it to a weaker LF inhibitor, the replacement of the hydroxamic acid group with the hydrolysis-resistant N, O-dimethyl hydroxamic acid (DHMA) group modestly improved the activity of LF inhibitors in cell culture [207]. The optimal type of chemicals to develop more compelling LF inhibitors remains to be determined [205].

Bacterial adhesion is an important step in pathogenesis, and is required for tissue colonization, where the next step of tissue destruction by enzymes and toxins takes place [208]. In fact, adhesion depends extensively on the surface properties of a bacterial cell, such as adhesion pili, which mediate cell–cell and cell–host attachments. These pili, also called fimbriae, are hair-like polymers composed of subunits assembled via sophisticated micro-machinery, and anchored in the bacterial outer membrane. Commonly, these pili are essential for the initiation of disease, and thus provide a potential target for antibacterial prevention and treatment [209]. Pili are important virulence factors in numerous bacterial pathogens, including *E. coli*, *Salmonella*, *Yersinia*, *Haemophilus*, *Pseudomonas*, and *Klebsiella*. Agents targeting bacterial adherence through the inhibition of pili not only deny access to host tissues, but they also promote rapid bacterial clearance and circumvent the release and translocation of tissue-damaging factors [177]. A group of bicyclic 2-pyridones, termed pilicides, were previously evaluated in uropathogenic *E. coli*. Hemagglutination mediated by either type 1 or type P pili, adherence to bladder cells, and biofilm formation mediated by type 1 pili were all reduced by approximately 90% in laboratory and clinical isolates. This represented promising evidence for developing drugs that function by targeting pili [210].

Two-component systems (TCS) for signal transduction are primary pathways by which bacteria adapt to the external environment, and they serve an important role in regulating virulent gene expression, cell wall synthesis, biofilm formation, and bacterial activity [211]. TCS allow organisms to sense and carry out a rapid physiological response to change in their environment, and are also implicated in antibiotic resistance, via the control of genes involved in antibiotic modification, the modification of antibiotic targets, and the biosynthesis of efflux pumps to remove the antibiotic outside the pathogen cell [212]. The proteins comprising TCS are the sensor histidine kinase (HK) and the response regulator (RR). These two factors are among the most abundant proteins that are widely distributed across bacteria [213]. In a prototype TCS, the HK and RR connect the detection of an environmental or cellular signal with an appropriate cellular response. Communication between the proteins is realized via phosphoryl group transfer from a histidine residue of the HK to an aspartate residue of the RR [214]. With such complex control of virulence by TSC, it is possible that the inhibitors of bacterial TCS can block the pathogenesis of important bacterial infective agents.

Most small-molecule inhibitors of TCSs target the HK. Several HK inhibitors have broad-spectrum activity and inhibit both essential and nonessential TCS. A small number of recently reported compounds are targeted to the RR, allowing for greater direct control over gene expression [212]. As such, several examples of small-molecule inhibitors that inhibit TCS exist. For example, dephostatin is a non-antibiotic compound that disrupts signaling through both the SsrA-SsrB and PmrB-PmrA TCS in *Salmonella*, and restores sensitivity to the last-resort antibiotic, colistin. Cellular-based and mouse models of infection demonstrate that dephostatin attenuates *Salmonella* virulence both in vitro and in vivo, through the perturbation of its regulatory networks [214]. In *S. aureus*, the small-molecule chemical savirin was capable of blocking the transcriptional function of the TCS Agr-CA, affecting virulence through the reduction of the quorum sensing ability, but not survival, while savarin had no impact on the commensal *Staphylococcus epidermidis* [215]. Another potent inhibitor of Agr-CA is staquorsin, recently described by Mahdally and colleagues [216]. In *P. aeruginosa*, benzothiazole-based HK inhibitors that perturb multiple virulence pathways were found to significantly reduce the level of toxic metabolites of this organism that are involved in quorum-sensing and redox-balancing mechanisms. The inhibitors also decrease the ability of *P. aeruginosa* to undergo swarming and attachment to surfaces, probably by influencing motility appendages [217]. Another inhibitor that reduces Enterobacteriaceae pathogen virulence in vivo is LED209. Studies measuring pathogen growth and survival in vitro and in infection models suggest that LED209 can reduce pathogenicity without affecting bacterial growth, via its effect on the TCS QseBC [218]. Recently, such inhibition was also studied in the reduction of the virulence of colitis-associated bacteria, including adherent-invasive *E. coli* (AIEC) [219]. Such inhibitors, therefore, are conquering gaining attention as antivirulence agents. 

Antivirulence therapies represent a strong, non-traditional trend towards the discovery and development of new antimicrobial agents; however, challenges remain to be addressed. Virulence factors are often species or even strain-specific, and variably conserved among a bacterial species. This makes their development specific for targeting particular pathogens. Furthermore, the expression of virulence factors may depend on environmental conditions, the site of infection, or the time course of a pathophysiologic process. Such complex biological variability and narrow spectrum constitute major challenges to translating the early discovery of virulence factors for clinical use [220].

### 6.4. Genome Mining

Genome mining refers to the bioinformatics investigation used to detect the biosynthetic pathway of bioactive natural products, as well as their possible functional and chemical interactions [221]. It is a process almost entirely dependent on bioinformatics tools and computing technologies [222]. The propensity of a microbial strain to produce molecules with novel chemical structures that could have new mechanisms of action in bacterial growth inhibition can be screened by this method, and the potential can be harnessed; with a focus on engineering the expression of the silent biosynthetic gene clusters predicted to encode novel antibiotics [223]. Currently, huge amounts of data, including DNA sequences and their annotations, are deposited in publicly accessible databases, with storage possible thanks to the development of computers and networks. Once all the genes within a genome are identified, they can be compared with those of known functions in the public databases. Both raw and annotated genomic data, as well as bioinformatics tools for sequence comparisons, are freely available [222]. The exploitation of natural product biosynthetic pathways via genome mining approaches is thus a prospect for the discovery of novel natural products with antibacterial properties, and relieves the situation of antimicrobial resistance.

For example, a new lichen-associating *Streptomyces* sp. YIM 130001 from a tropical rainforest in China displayed antibacterial activity against *Bacillus subtilis*. The draft genome sequence of this isolate strain revealed 18 putative biosynthetic gene clusters (BGCs) for secondary metabolites. An analysis of these gene clusters led to the identification of new thiopeptide antibiotic, geninthiocin B, with bioactivity against *B. subtilis*. A combined analysis of the genome sequencing data and metabolite profiling led to the identification of the geninthiocin B gene cluster, confirming the power of the genome mining approach in the discovery of natural products with antibacterial activity [224]. Li and colleagues [225] analyzed over 7300 bacterial genomes to investigate their capacity for the biosynthesis of cationic nonribosomal peptides with activity against Gram-negative bacteria. They were able to identify two novel compounds, brevicidine and laterocidine, with bactericidal activities against *P. aeruginosa* and colistin-resistant *E. coli*, and an apparently low risk of resistance. The two peptides showed efficacy against *E. coli* in a mouse thigh infection model, contributing to a novel pathway in the discovery and development of antibiotics against Gram-negative pathogens.

### 6.5. Microbiome-Modulating Agents

Humans host trillions of micro-organisms in the skin, gut, and airways. These micro-organisms exert regulatory functions, are in a continuous dialogue with intestinal epithelia, influence energy production, break down nutrients, digest vitamins, and may interfere with the pathophysiology of diabetes and obesity [226]. Short-term antibiotic treatment shifts the gut microbiota to long-term alternative dysbiotic states, which may promote the development and aggravation of disease. Common features of such post-antibiotic dysbiosis are a loss of taxonomic and functional diversity, and a reduced colonization resistance against invading pathogens [227]. As such, the modulation of the gut microbiome may prove to be a useful strategy to reduce the influence of changes induced by antibiotics in the gut. Microbiome-modulating agents are currently in clinical trials. A synthetic glycan is being investigated to enhance the growth of beneficial gut microbes to boost immune response. There are also antibiotic inactivators in clinical trials that help maintain gut microbes by either the degradation of excess penicillin and cephalosporins in the gut, or by using activated charcoal to absorb excess antibiotics and their degradative metabolites to better preserve the intestinal microbiota [11].

### 6.6. Antibacterial Antibodies

Despite the explosion in monoclonal antibody therapies developed for oncologic and rheumatic indications, the therapeutic potential of this drug class for treating multidrug-resistant infections is yet to be examined [228]. Given the complexity of bacterial pathogenesis processes, antibody therapeutics are expected to be efficient upon targeting more than one virulence factor and/or combining different modes of action. An improved understanding of bacterial pathogenesis, combined with the versatility and maturity of antibody discovery technologies, are both instrumental for the design of novel anti-bacterial therapeutics [229]. For example, antibody-based biologics are being studied to target *S. aureus* [230], and a monoclonal antibody targeting protein A of this organism was found to be promising in protection against sepsis and peritonitis in mice [231]. Likewise, anticapsular antibodies were found to be protective against the most virulent carbapenem-resistant *K. pneumoniae* clinical strains. These antibodies promoted bacterial killing through several extracellular and intracellular processes, and prevented the spread of infection in mice from the lungs to distal organs. It is promising that such antibodies can ultimately treat or protect patients infected or at risk of infection by this multidrug-resistant bacterium [232]. It is evident that intensified research and the increasing number of clinical programs with anti-bacterial antibodies shall render this field ready to fulfill its potential in the coming years when new therapeutic targets for antibiotic-resistant bacterial pathogens are desperately needed [229].

It is worth mentioning that, in addition to the above, the preclinical antibiotic pipeline holds 135 projects on direct-acting small molecules that represent new classes, new targets, or new mechanisms of action, with a vast number of pathogen-specific and adjunctive approaches not witnessed before in antibiotic discovery, as reported recently in 2020 [13]. The innovative potential of the preclinical pipeline compared with the clinical pipeline is encouraging. However, despite promising signs, more work, more funding, and more focus are required to translate scientific advances into clinically approved new antibiotics [168]. 

## 7. Antibiotics Research and Development: Incentives and Barriers

### 7.1. Incentives to Research and Development

The discovery and development of new antibiotics take at least ten years and cost more than USD 1 billion [233]. In parallel, the development and spread of AMR take an alarmingly short amount of time and race forwards as soon as the marketing of the antibiotic starts. As a result, companies are shifting research and development (R&D) away from investing in the antibiotic pipeline [3,234]. Estimates have shown that out of USD 38 billion venture capital funds invested in R&D between 2003 and 2013, only USD 1.8 billion were allocated in antimicrobial research and development [235]. Antibiotic discovery and development are determined based on government priorities, industry demands, and other operational realities specific to each country [235]. The scientific and economic challenges are huge [12]. Funding gaps stem from the early stages of screening and hit compounds (molecules that show a desired type of activity in an initial screening assay(s)) generation, as well as hit-to-lead and lead optimization stages led by academia and by small and medium-sized enterprises (SMEs) [12]. To identify novel hits and leads and nominate pre-clinical candidates, it may take from 1 to 7 years of research associated with a cost 5–10 million Euros, excluding the costs for attrition and lost opportunities of the increased cycle time to reach the next development phase [12,236]. In other terms, SMEs and the academic sector are driving innovation, since large companies are dropping out of the field [9]. This implies that SMEs are facing, with limited capacity, the risks and down-turns of product failure in the early stages, despite the fact that this research stage is the mainstay in R&D, to ensure the sustainability of new antibacterial drug candidates in the development pipeline. The main funding programs are focusing on the pre-clinical stages as a result of private–public partnerships, such as the Global Antibiotic Research and Development Partnership (GARDP), Combating Antibiotic-Resistant Bacteria Biopharmaceutical Accelerator (CARB-X) [160,237,238], Innovative Medicines Initiative (IMI), and Drugs for Neglected Diseases Initiative (DNDi), in addition to international as well as national non-profit organizations and government programs [12]. Multiple incentives are available to boost R&D initiatives, which may or may not be efficient, depending on the context and framework. A balanced push and pull incentives mechanism is needed to select an effective package that addresses the core market failures. The push incentives directly support R&D, while the pull incentives reward successful outcomes from R&D. The push incentives do not improve the attractiveness of the overall market. The pull incentives are needed to attract the private sector funding and avoid relying only on public and philanthropic sources [239]. The research project “Driving reinvestment in research and development for antibiotics and advocating their responsible use” (DRIVE-AB) assessed more than 30 different incentives from different industries, intending to change the way in which policymakers stimulate innovation and ensure the sustainable use of antibiotics [240,241]. This project was a consortium of joint efforts between public sector partners and pharmaceutical companies supported by the European IMI. DRIVE-AB determined the four most effective push incentives in stimulating the antibiotic pipeline such as (1) grants given to academia, companies, and other; (2) pipeline coordinators that are non-governmental or non-profit organizations that track the antibiotic pipeline, identify gaps, and support R&D financially and technically; (3) market entry rewards for successfully achieving regulatory approval for an antibiotic that meets specific predefined criteria for the benefit of the public; and (4) the long-term supply continuity model, a delinked payment to create a predictable supply of generic antibiotics [241]. Each incentive plays a role throughout the drug development process for SMEs involved in antibiotic R&D, and is considered the engine for discovery and early development [241,242]. The government can offer the firms incentives to avoid profit-driven promotions and reduce overproduction to maximize profits such as patency, monopoly, and property rights, to allow the exclusive use of their products for a determined period [132]. Pharmaceutical companies tend to overproduce and oversell their product to maximize their profit and increase the return on investment before the patency expires [243]. Extending the patency for a defined time frame is thought to incentivize pharmaceutical companies to preserve antibiotic effectiveness for a prolonged period [244]. Monopoly is an additional potential solution that boosts economic efficiency by internalizing the societal cost through giving the pharmaceutical company an incentive to self-regulate and promote the conservation of antibiotic effectiveness for long-term profits [243]. If monopoly gives the firm control over one antibiotic, the problem of cross-resistance should be considered [245]. The ideal strategy is giving the pharmaceutical company full control to produce and distribute all antibiotics on the market [244], which is impossible. Despite the creation of public/private initiatives, the push and pull incentives remain focused on the economic return [246]. Determining which ones, and if antibiotic incentive strategies are the most effective to revitalize the antibiotic pipeline, is crucial, as incentives may be country-specific and associated with multiple advantages and disadvantages depending on the funded entity [9].

### 7.2. Challenges to Antibiotic Discovery

The challenges for antibiotic discovery are primarily financial, complicated by the scientific and regulatory hurdles [247,248]. The high probability of attrition has a tremendous influence on the milestone of antibiotic discovery, and difficulty finding innovative new leads [248]. The uncertainty has led pharmaceutical companies to withdraw antibiotic development programs [226]. The big pharmaceutical industry left behind the canonical discovery of new antibiotics [228]. Since the 1980s, more than 25 pharmaceutical companies invested in antibiotic discovery dropped from R&D, and very few have ongoing internal research [223]. The exodus of big pharma combined with the shrinkage in the pool of scientific experts in the field has shifted the pressure of research to academia and SMEs already struggling with limited expertise to lead research programs, the potential financial impact of lead failure, and the lack of funding of clinical trials. Other challenges of new antibiotic discovery are related to redundancy [9,248,249], where new drugs in the clinical pipeline or recently marketed are improvement or the modification of old antibiotics characterized by broader bacterial spectrum and improved simple dosage regimens, and safety margin [229].

The other huddle is related to strict regulations for clinical trials. Some drugs with improved activity against resistant pathogens such as oritavancin were pushed back due to the inadequacy in trials. The pharmacological and toxicological criteria are also challenging in repurposing original drugs and their starting material [227].

## 8. Future Perspectives

A lot remains to be investigated to tackle the future of AMR, augment antibiotic discovery, and enlarge the armamentarium of agents used to combat MDR pathogens. The different lessons learned from the golden age of antibiotic discovery, changes in AMR thereafter, and consideration of the current recent advances in genome editing, gene regulation, and systems biology, can all inspire a wide variety of innovative antibiotic discovery projects. Approaches including nanoparticles, immunotherapy, antisense RNA, resistance modulation, and removal of drug-resistance plasmids are on the rise. Moreover, antibiotic resistance breakers that may or may not have direct antibacterial action and can either be co-administered with or conjugated to other failing antibiotics, are being studied. In this regard, compounds that are used to reverse AMR like modifying-enzyme inhibitors, membrane permeabilisers and efflux pump inhibitors are under investigation. Other possible future avenues to combat AMR include antibiotic hybridization, harvesting, and modifying natural antimicrobial peptides from eukaryote and prokaryote organisms [250]. Although for the foreseeable future, it is likely that most of these approaches, if approved, will be used in combination with antibiotics, they still represent hope towards promising methods to combat AMR [220].

## 9. Conclusions

A throwback on the history of antibiotics and the evolution of resistance shows that, in a matter of about one century, antibiotics have drastically changed modern medicine and increased human lifespan, however, have been also substantially hit by the current AMR crisis. This is a multifactorial issue of microbial evolution to escape antibiotics, gradual decline in discovery and development of antibiotics, and many economic and societal challenges. The race between AMR and antibiotic discovery shall continue, and research needs to chase, at higher speeds, and with more prominence an intensity, augmented solutions to combat AMR. While the global efforts to mobilize attention towards AMR have been huge, the call today is to further invest in basic, translational, and clinical research targeting AMR, and to optimize the application of advanced knowledge and expertise to target this public health concern in humans and the environment.

## Figures and Tables

**Table 1:** The WHO and CDC listing of priority pathogens and the level of threat.

	WHO [134]		CDC [135]	
Organisms	Pathogens	Level of Priority	Pathogens	Level of Threat
*Acinetobacter baumannii*	*Acinetobacter baumannii*, carbapenem-resistant	Critical		Urgent *
*Pseudomonas aeruginosa*	*Pseudomonas aeruginosa*, carbapenem-resistant	Critical	Multidrug-resistant *Pseudomonas aeruginosa* ^1^	Serious **
*Enterobacteriaceae*	*Enterobacteriaceae*, carbapenem-resistant, ESBL-producing	Critical	*Enterobacteriacea*, Carbapenem-resistant ^2 3^	Urgent
	*Enterobacteriaceae*, ESBL-producing	Critical	Extended-spectrum β-lactamase (ESBL)-producing Enterobacteriaceae ^4^	Serious
*Enterococcus faecium*	*Enterococcus faecium*, vancomycin-resistant	High	Vancomycin-resistant Enterococci (VRE)	Serious
*Staphylococcus aureus*	*Staphylococcus aureus*, methicillin-resistant	High	Methicillin-resistant *Staphylococcus aureus* (MRSA) ^5^	Serious
*Staphylococcus aureus*	*Staphylococcus aureus*, vancomycin-intermediate and resistant	High		
*Helicobacter pylori*	*Helicobacter pylori*, clarithromycin-resistant	High		
*Campylobacter* spp.	*Campylobacter* spp., fluoroquinolone-resistant	High	Drug-resistant *Campylobacter*	Serious
*Salmonellae*	*Salmonellae*, fluoroquinolone-resistant	High	Drug-resistant nontyphoidal *Salmonella* Drug-resistant *Salmonella* serotype Typhi	Serious
*Neisseria gonorrhoeae*	*Neisseria gonorrhoeae*, cephalosporin-resistant, fluoroquinolone-resistant	High	Drug-resistant *Neisseria gonorrhoeae*	Urgent
*Streptococcus pneumoniae*	*Streptococcus pneumoniae*, penicillin-non-susceptible	Medium	Drug-resistant *Streptococcus pneumoniae*	Serious
			Erythromycin-Resistant Group A *Streptococcus* Clindamycin-resistant Group B *Streptococcus*	Concerning ***
*Haemophilus influenzae*	*Haemophilus influenzae*, ampicillin-resistant	Medium		
*Shigella* spp.	*Shigella* spp., fluoroquinolone-resistant	Medium	Drug-resistant *Shigella*	Serious
*Clostridioides difficile*				Urgent
*Mycobacterium tuberculosis*	Not listed in the 2017 high priority pathogens because it is previously established as high priority		Drug-resistant tuberculosis	Serious
*Bordetella pertusis*			Drug-resistant *Bordetella pertusis*	Watch

* These germs are public health threats that require urgent and aggressive action; ** These germs are public health threats that require prompt and sustained action; *** These germs are public health threats that require careful monitoring and prevention action. 1—Extended-spectrum cephalosporins (cefepim, ceftazidime); Fluoroquinolones (ciprofloaxacin, levofloxacin); Aminoglycosides (amikacin, gentamicin, tobramycin); Carbapenems (imipenem, meropenem, doripenem); Piperacillin Group (piperacillin, piperacillin/tazobactam). 2—*E. coli*, *Klebsiella* spp., *Enterobacter* spp. 3—Imipenem, meropenem, doripenem, ertapenem, ampicillin, ampicillin/sulbactam, amoxicillin/clavulanic acid, piperacillin/tazobactam, cefazolin, cefoxitin, cefotetan. 4—Cefotaxime, ceftriaxone, ceftazidime, cefepime, ampicillin, piperacillin, aztreonam, cefazolin. 5—Methicillin, oxacillin, cefoxitin.

**Table 2:** A representation of current candidate antibacterial products in current phases of development with their classes, expected activity against pathogens, expected administration route, and innovativeness.

Product Name	Alternative Name	Product Type		Non-Traditional Categories					R&D Phase				Antibacterial Class	Expected Activity against Priority Pathogens														Route of Administration			Innovative
														Critical Priority Pathogens				Other Priority Pathogens										IV	Oral	Inh	
		Non-traditionals	Antibiotics	Antibodies	Microbiome modulating agents	Bacteriophages and phage-derived enzymes	Immunomodulating agents	Miscellaneous	Phase I	Phase II	Phase III	Unknown		*Acinetobacter baumannii*	*Pseudomos aeruginosa*	*Enterobacterales*	All critical priority pathogens	Gram-positive priority pathogens	*Neisseria gonorrhea*	*Helicobacter pylori*	*Staphylococcus aureus*	*Enterococcus faecium*	*Streptococcus pneumoniae*	*Campylobacter* species	Other priority pathogens	*Mycobacterium tuberculosis*	*Clostridium difficile*				
514G3	True human™ Mab	•		•						•			Anti-*Staphylococcus aureus* IgG monoclonal Ab					•			•							•			
AB103	Reltecimod	•					•				•		Antagonist of superantigen exotoxins and CD28 T-cell					•			•				•			•			
ACX-362E	-		•							•			D polymerase IIIC inhibitor														•		• (na)		•
Afabicin	Debio-1450		•							•			FabI inhibitor	No	No	No	No	•	No	No	•	No	No	No	•			•	•		•
AR-101	Panobacumab, Aerumab	•		•						•			Anti-*Pseudomonas aeruginosa* serotype O11 IgG monoclonal Ab		•													•			
AR-105	Aerucin	•		•						•			Anti-*P. aeruginosa* IgG1 monoclol Ab		•													•			
AR-301	Tosatoxumab	•		•							•		Anti-*S. aureus* IgM monoclol Ab					•			•							•			
ARX-1796	Oral Avibactam prodrug		•						•				DBO-BLI + β-lactam	No	No	•	No	No	No	No	No	No	No	No	No				•		No
Bepenem	-		•							•			Carbapenem	No	No	No	No											•	•		No
BT588	Trimodulin	•		•						•			Anti-*S. aureus* polyvalent Ab (IgM, IgA and IgG)					•			•							•			
BTZ-043	-		•							•			DprE1 inhibitor (benzothiazinone)													•			•		•
CAL02	-	•						•	•				Broad spectrum anti-toxin liposomal agent and noparticle					•			•							•			
CF-301	Exebacase	•				•					•		Phage endolysin					•			•							•			
CP101	–	•			•					•			Live biotherapeutic product														•		•		
CRS3123	-		•						•				Methionyl-tR synthetase inhibitor (MetRS)														•		•		•
DAV132	–	•			•					•			Antibiotic ictivator and protective colon-targeted adsorbent														•		•		
Delpazolid	LCB01-0371		•							•			Oxazolidinone													•			•		No
DNV-3827	MCB-3837		•							•			Oxazolidinone-quinolone hybrid														•	•			Inconclusive
DSTA4637S	RG7861	•		•					•				Anti-*S. aureus* IgG mAb/rifamycin					•			•							•			
Durlobactam + sulbactam	ETX-2514		•								•		DBO-BLI /PBP2 binder + β-lactam-BLI/PBP1,3 binder	•	No	No	No											•			No
EBL-1003	Apramycin		•						•				Aminoglycoside	Pos	No	Pos	No											•			No
Enmetazobactam + cefepime	AAl-101 + cefepime		•								•		β-lactam BLI + cephalosporin	No	No	No	No											•			No
ETX0282 + cefpodoxime	–		•						•				DBO-BLI/PBP2 binder + cephalosporin	No	No	•	No												•		No
Ftortiazinon + cefipime	Fluorothyazinone	•						•		•			Type III secretion system inhibition + cefepime		•														•		
Gepotidacin	–		•								•		Topoisomerase inhibitors (Triazaacephthylene)		No			•	•	No	•	No	No	No	•			•	•		•
GSK-3036656	GSK-070		•							•			Leu RS inhibitor (oxaborole)													•			•		•
GSK3882347	-	•						•	•				FimH antagonist			•													•		
IM-01	–	•		•						•			FimH antagonist														•		•		
KB109	-	•			•							•	Anti-*Clostridium difficile* polcyclonal Ab			•						•			•				•		
KBP-7072	–		•						•				Tetracycline	•	No	No	No	•	No	No	•	No	No	No	•				•		No
LBP-EC01	–	•				•			•				CRISPR-Cas3 enhanced phage			•												•			
LMN-101	–	•		•						•			Monoclol Ab-like recombinant protein			•								•	•				•		
Macozinone	PBTZ-169		•							•			DprE1 inhibitor (Benzothiazinone)													•			•		•
MEDI-4893	Suvratoxumab	•		•						•			Anti-*S. aureus* IgG monoclonal Ab					•			•							•			
MET-2	-	•			•				•				Live biotherapeutic product														•		•		
MGB-BP-3	–		•							•			D minor groove binder (distamycin)														•		• (na)		•
cubactam + meropenem	–		•						•				DBO-BLI/PBP2 binder + cephalosporin	No	No	•	No											•			No
fithromycin	WCK-4873		•							•			Macrolide					•	No	No	•	No	•	No	•				•		No
OligoG	CF-5/20	•						•		•			Algite oligosaccharide (G-block) fragment		•															•	
OPC-167832	–		•							•			DprE1 inhibitor (3,4-dihydrocarbostyril)													•			•		•
Phage Bank	–	•				•				•			Phage bank (process)			•													•		
PLG0206	WLBU2		•						•				Cationic peptide	Pos	Pos	Pos	Pos	•			•				•			•			•
QPX7728 + QPX2014	-		•						•				Borote-BLI + unknown	•	Pos	•	Pos											•			Inconclusive
RBX7455	-	•			•				•				Live biotherapeutic product														•		•		
Rhu-pGSN	Rhu-plasma gelsolin	•					•			•			Recombint human plasma gelsolin protein	•	•	•	•	•	•	•	•	•	•	•	•			•			
Ridinilazole	–		•								•		Bis-benzimidazole														•		• (na)		•
SAL-200	Tobacase	•				•				•			Phage endolysin					•			•							•			
SER-109	-	•			•						•		Live biotherapeutic product														•		•		
SPR-206	-		•						•				Polymyxin	•	•	•	•											•			No
SPR-720	-		•							•			GyrB inhibitor (benzimidazole ethyl urea)													•			•		•
Sulopenem, sulopenem etzadroxil/probenecid	–		•								•		Penem	No	No	No	No											•	•		No
Sutezolid	–		•							•			Oxazolidinone													•			•		No
SYN-004	Ribaxamase	•			•					•			Antibiotic inactivator														•		•		
Taniborbactam + cefepime	VNRX-5133 + cefepime		•								•		Borote-BLI + cephalosporin	No	Pos	•	No											•			•
TBA-7371	–		•							•			DprE1 inhibitor (azaindole)													•			•		•
TBAJ-876	-		•						•				Diarylquinoline													•			•		No
TBI-166	–		•						•				Riminophezine (clofazimine-alogue)													•			•		No
TBI-223	-		•						•				Oxazolidinone													•			•		No
Telacebec	Q-203		•							•			Imidazopyridine amide													•			•		•
TNP-2092	–		•							•			Rifamycin-quinolizinone hybrid	No	No	No	No	Pos	No	Pos	Pos	Pos			Pos			•	•		No
TNP-2198	-		•						•				rifamycin-nitroimidazole conjugate					No	Pos	•	No				•				•		No
TP-271	–		•						•				Tetracycline	Pos	No	No	No	•	No	No	•	•	No	No	•			•	•		No
TP-6076	–		•						•				Tetracycline	•	No	Pos	No											•			No
TXA709	-		•						•				FtsZ inhibitor	No	No	No	No	•	No	No	•	No	No	No	•			•	•		•
VE303	–	•			•					•			Live biotherapeutic product														•		•		
VNRX-7145 + ceftibuten	–		•						•				Borote-BLI + cephalosporin	No	No	•	No												•		•
Zidebactam + cefepime	–		•						•				DBO-BLI/ PBP2 binder + cephalosporin	•	•	•	•											•			No
Zoliflodacin	–		•								•		Topoisomerase Inhibitors (Spiropyrimidenetrione)	No	No	No	No	No	•	No	No	No	No	No	•				•		•

Table was adapted from the World Health Organization Global Observatory on Health Research and Development; section on antibacterial products in clinical development for priority pathogens; April 2021. Innovative: meets all four following criteria: new chemical class, new target or binding site, new mode of action, and/or no cross resistance to other antibiotic classes; na: Not absorbed; Pos: Possibly; Inh: Inhalation.
